# Records of *Parochlus steinenii* in the Maritime Antarctic and sub-Antarctic regions

**DOI:** 10.3897/zookeys.1011.56833

**Published:** 2021-01-18

**Authors:** Melisa Gañan, Tamara Contador, Javier Rendoll, Felipe Simoes, Carolina Pérez, Gillian Graham, Simón Castillo, James Kennedy, Peter Convey

**Affiliations:** 1 Wankara Sub-Antarctic and Antarctic Freshwater Ecosystems Laboratory, Sub-Antarctic Biocultural, Conservation Program, Universidad de Magallanes, Puerto Williams, Teniente Muñoz 166, Chile Universidad de Magallanes Puerto Williams Chile; 2 Institute of Ecology and Biodiversity, Universidad de Chile, Santiago, Las Palmeras 3425, Chile Núcleo Científico Milenio Concepción Chile; 3 Núcleo Milenio de Salmónidos Invasores (INVASAL) Iniciativa Científica Milenio, ICM, Núcleo Científico Milenio, Concepción, Chile Universidad de Chile Las Palmeras Chile; 4 British Antarctic Survey, NERC, High Cross, Madingley Road, Cambridge CB3 0ET, UK British Antarctic Survey, NERC Cambridge United Kingdom; 5 Department of Zoology, Museum of Zoology, University of Cambridge , Downing Street, Cambridge CB2 3EJ, UK University of Cambridge Cambridge United Kingdom; 6 Department of Biological Sciences, University of North Texas, 1511W Sycamore, Denton, TX 76201, USA University of North Texas Denton United States of America; 7 Department of Ecology, Pontificia Universidad Católica, Facultad de Ciencias Biológicas. Avda. Libertador Bernardo O’Higgins 340, Santiago, Chile Pontificia Universidad Católica Santiago Chile

**Keywords:** Cape Horn Biosphere Reserve, *Parochlus steinenii*, South Georgia, South Shetland Islands, winged Antarctic midge

## Abstract

This study provides the summary of the reports of the geographical distribution in the Maritime Antarctic and sub-Antarctic regions of *Parochlus steinenii* (Gercke, 1889) (Diptera, Chironomidae), the only flying insect occurring naturally in the Antarctic continent. The distribution encompasses the South Shetland Islands (Maritime Antarctic), South Georgia (sub-Antarctic), and parts of the Cape Horn Biosphere Reserve (CHBR, southern Chile). In total 78 occurrence records were identified, 53 from our own records, 19 from the literature, and six from other data present in GBIF. Of the 78 records, 66 are from the South Shetland Islands, eight are from South Georgia, and four from the CHBR. This database was developed as one of the main objectives of two Chilean-funded research projects addressing understanding the effects of climate change on sub-Antarctic and Antarctic insects. It provides dataset documenting the distribution of *Parochlus steinenii* in the Maritime Antarctic, the sub-Antarctic, and the CHBR in southern South America (Chile). The complete dataset is available in Darwin Core Archive format via the Global Biodiversity Information Facility (GBIF).

## Project details

**Project title**: This database was developed as part of the main objectives of two Chilean-funded research projects aiming towards better understanding the effects of climate change in sub-Antarctic and Antarctic insects:

- Dipterans in sub-Antarctic and Antarctic regions: are they ready for the changes?
- Addressing global warming scenarios in freshwater ecosystems using aquatic insects as model organisms in the Magellanic sub-Antarctic and Antarctic regions.

**Personnel**: Melisa Gañan (Data Collector, Data Manager, Data Publisher), Tamara Contador (Principal Investigator, Data Collector, Data Manager, Data Publisher), Javier Rendoll (Data Collector, Data Manager), Felipe Simoes (Data Collector, Data Manager), Carolina Pérez (Data Collector, Data Manager), Gillian Graham (Data Collector), Simón Castillo (Data Collector), James Kennedy (Data Collector, Data Manager) and Peter Convey (Data Collector, Data Manager).

**Funding**: INACH RT-48_16 and Fondecyt de Iniciación 11130451.

**Figure 1.**
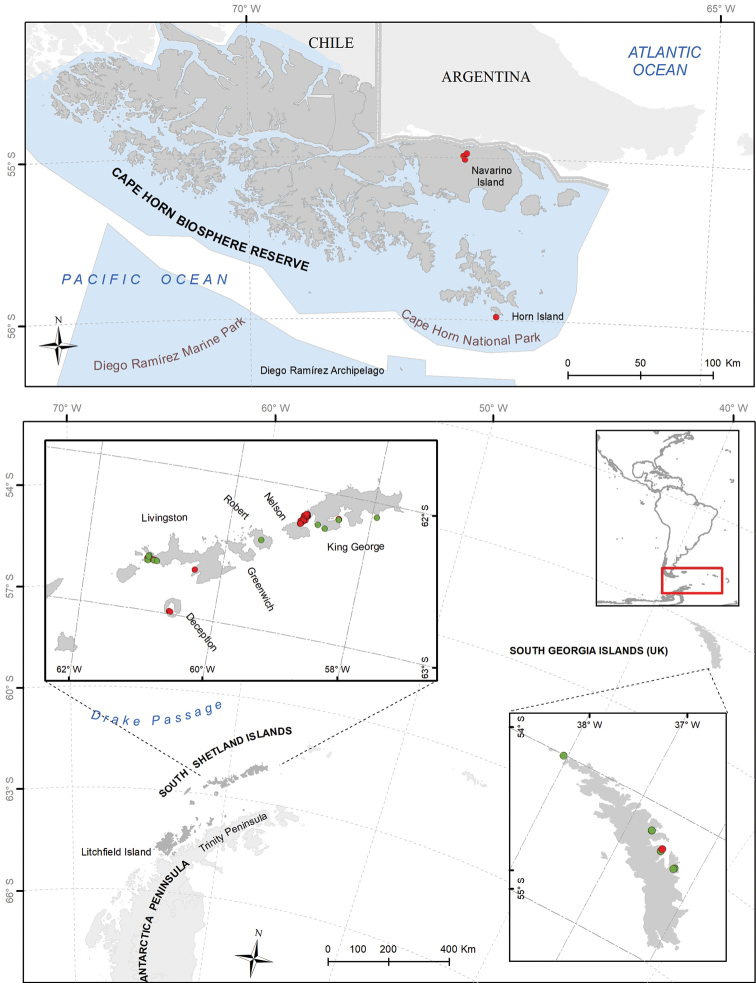
Map of study area. The red circles correspond to the records of *Parochlus steinenii* found in this study, the green circles correspond to the bibliographic records.

**Study area descriptions/descriptor**: The study area (Fig. 1) includes: 1. The South Shetland Islands and part of the north-west coast of the Antarctic Peninsula (Antarctic Conservation Biogeographic Region (ACBR) 3, Terauds et al. 2012; Terauds and Lee 2016); 2. The sub-Antarctic island of South Georgia; 3. Navarino Island, Cape Horn National Park, and Diego Ramírez Marine Park (Cape Horn Biosphere Reserve, Magallanes sub-Antarctic region, Rozzi et al. 2012).

**Design description**: The study was conducted throughout the latitudinal and environmental gradient that includes the southern tip of South America in the sub-Antarctic Magellanic ecoregion (54–57°S), and the Scotia Arc distribution of *Parochlus steinenii* (*P. steinenii*) in the sub-Antarctic (South Georgia, 53–54°S) and Maritime Antarctic (South Shetland Islands, 63–64°S) regions. The geographical range of the study involves both small-scale (microhabitats and environmental gradients) and the larger spatial scale 10-degree latitudinal gradient.

The specific locations surveyed were: 1. On the maritime Antarctic South Shetland Islands, ice-free areas on Deception, Livingston, Greenwich, Robert, Nelson, King George Islands, and the north-west coast of Antarctic Peninsula (Trinity Peninsula and Litchfield Island). These areas are characterized by a geomorphology which includes periglacial landforms, with numerous temporary shallow meltwater ponds and permanent lakes (typically smaller than 100 m²), which are ice-covered for 9–10 months of the year (Contador et al. 2020); 2. In South Georgia, which is the largest island on the Scotia Arc, with much of the barren, mountainous and highly glacial territory (Regional ecosystem profile – Polar and Sub-polar Region 2017), we surveyed accessible lakes near the British Antarctic Survey research station; 3. In CHBR, we surveyed the north coast of Navarino Island, with freshwater systems that present altitudinal gradients with marked slopes in an altitudinal range that extends between 1000 m and sea level, and where the thermal ranges change drastically along of the altitudinal profile (Contador 2011; Contador et al. 2015). We also surveyed Horn Island in the southernmost of the Cape Horn archipelago, and the Diego Ramirez archipelago, both made up of low-lying islets and marked by strong winds throughout the year (Pisano 1980, Pisano and Schlatter 1981).

Field expeditions were organized by the Chilean Antarctic Institute (INACH) in South Shetland Islands; by the British Antarctic Survey in South Georgia; and by the Sub-Antarctic Biocultural Conservation Program of the Universidad de Magallanes in CHBR.

## Taxonomic coverage

**General taxonomic coverage description**: The dataset reports occurrences of the species *Parochlus steinenii* (Gercke, 1889) (Diptera: Chironomidae: Podonominae), also known as the winged Antarctic midge. *P. steinenii* is recorded from freshwater ecosystems through the ice-free areas of the South Shetland Islands in the Maritime Antarctic, on sub-Antarctic South Georgia, and in Navarino and Horn islands in the CHBR. Knowing these presences is of great importance since *P. steinenii* has been proposed as an effective native sentinel species and indicator of climate change in Antarctica (Contador et al. 2020).

### Taxonomic ranks

**Kingdom** Animalia

**Phylum** Arthropoda

**Class** Insecta

**Order** Diptera

**Family** Chironomidae

**Subfamily** Podonominae

**Tribe** Podonomini

**Genus** *Parochlus*

**Species** *Parochlus steinenii*

**Common name**: winged Antarctic midge

## Spatial coverage

**General spatial coverage**: The dataset comprises the South Shetland Islands, specifically King George, Nelson, Robert, Livingston and Deception islands in the Maritime Antarctic, South Georgia in the sub-Antarctic, and Horn and Navarino islands in the CHBR (southern South America, Chile).

**Coordinates**: The areas surveyed lie within the polygon 53–63°S latitude and 35–69°W longitude.

## Temporal coverage

January 1, 2014–February 25, 2019.

## Natural collections description

**Parent collection identifier**: UMAG: Wankara Laboratory

**Collection name**: Colección de Invertebrados Antárticos y Subantárticos del Laboratorio Dulceacuícola Wankara de la Universidad de Magallanes, Puerto Williams

**Collection identifier**: urn:UMAG:WANKARA:Inv:Dip:AQ:Pstei and urn:UMAG:WANKARA:Inv:Dip:CL:Pstei

**Specimen preservation method**: 95% alcohol

**Curatorial unit**: 20 individuals per 5 ml glass vials

## Methods

**Survey and sampling**: Intensive field surveys through accessible ice-free areas in the South Shetland Islands, through lakes on South Georgia, and lakes on Horn Island, Navarino Island, and Diego Ramírez archipelago were conducted. For each sample site, the presence of *P. steinenii* was assessed and reference collection of individuals was made, the macrohabitat was described, and the climatic and water variables were recorded. Survey data were combined with information from a careful bibliographic review.

**Figure 2.**
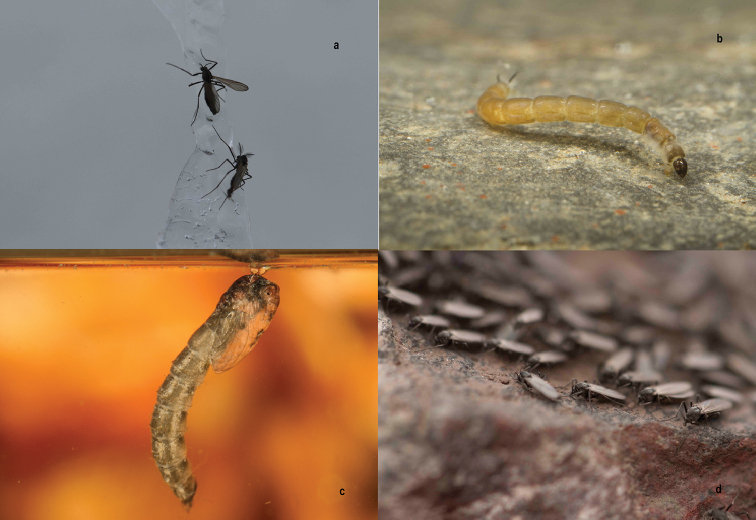
*Parochlus steinenii* (Fildes Bay, South Shetland Islands). **a** Female (up) and male (down) adult **b** larva **c** pupa **d** group of adults on a stone.

**Sampling description**: All sites were sampled for a period of 4–6 h, depending on climatic conditions and logistic support. We assessed the presence of *P. steinenii* as larvae, pupae, or adults by searching close to the shoreline of lakes and streams, and specifically under rocks and vegetation, and in sediments (Fig. 2). We manually extracted specimens from rocks and mosses and sometimes with the use of an entomological aspirator. Each site visited was georeferenced using a Garmin 78SC GPS receiver. Water body typology and macrohabitat were described following Hahn and Reinhardt (2006). Climatic variables (air temperature, wind speed, and relative humidity) were measured using an anemometer (Kestrel 3000 Env) and water variables (pH, conductivity, water temperature, and dissolved oxygen) were measured using a YSI 605 595 Professional Plus multimeter. We additionally sourced all available information from the existing literature (see Torres 1956; Wirth and Gressitt 1967; Brundin 1970; Edwars and Usher 1985; Rauschert 1985; Shimada et al. 1991; Richard et al. 1994; Convey and Block 1996; Allegrucci et al. 2006; Hahn and Reinhardt 2006; Toro et al. 2006; Agius et al. 2008; Rico and Quesada 2013).

Fieldwork in the Antarctic was conducted during six austral summer seasons (2013/14, 2014/15, 2015/16, 2016/17, 2017/18 and 2018/19). In South Georgia fieldwork was conducted in the Austral summer of 2018 and in the Magellanic sub-Antarctic Region in the Austral summer of 2016.

Living individuals were transported to the laboratory for phenology and physiologic studies, while some individuals were immediately preserved in alcohol (95%) for genetic studies. Samples were transported to the Wankara Subantarctic and Antarctic Freshwater Studies Laboratory at Magallanes University in Puerto Williams, Chile. Characteristics of the species according to the taxonomic key of Wirth and Gressitt (1967) were verified in the laboratory. To date, the species was not recorded in any extensive terrestrial/freshwater study along the Diego Ramírez Island, South Orkney Islands, or north-west coast of the Antarctic Peninsula (Chown and Convey 2016; Contador et al. 2020).

**Quality control description**: Each record of the species obtained in the field was georeferenced using a Garmin 78SC GPS receiver. Most records obtained from literature included geographical coordinates. Otherwise, we assigned a georeference record by identification of the body of water described in the study.

Geographic names used for records presented here follow the official name used in the maps prepared by the SCAR Composite Gazetteer of Antarctica (CGA) and by the Military Geographical Institute (IGM) of Chile. For sites lacking formal names, unofficial names were assigned.

**Data resources**: The data set of this article is deposited at GBIF, the Global Biodiversity Information Facility, https://www.gbif.org/dataset/30c49fbf-4e2e-482e-bb49-4d294bc332cb, https://doi.org/10.15468/2cfwd7.

## Datasets

### Dataset description

**Object name**: Darwin Core Archive Presence Parochlus steinenii AQ_CL

**Character encoding**: UTF-8

**Format name**: Darwin Core Archive format

**Format version**: 1.0

**Distribution**: https://www.gbif.org/dataset/30c49fbf-4e2e-482e-bb49-4d294bc332cb

**Publication date of data**: 2020-05-27

**Language**: English

**Licenses of use**: Creative Commons Attribution (CC-BY) 4.0 License

**Metadata language**: English

**Date of metadata creation**: 2020-05-27

**Hierarchy level**: Dataset

## Records from other data published in GBIF

Hårsaker K, Aspaas AM, Dolmen D, Ekrem T, Munkeby TB, Stur E, Frode Ø, Aagaard Finstad AG (2020) Terrestrial and limnic invertebrates systematic collection, NTNU University Museum. Version 1.290. NTNU University Museum. Occurrence dataset. https://doi.org/10.15468/fsreqb [accessed via GBIF.org on 02.10.2020]

The International Barcode of Life Consortium (2016) International Barcode of Life project (iBOL). Occurrence dataset. https://doi.org/10.15468/inygc6 [accessed via GBIF.org on 02.10.2020]
